# A readily available surgical assistant

**DOI:** 10.1308/003588413X13511609958055a

**Published:** 2013-03

**Authors:** MJ Carr, AR Day, PC Hale

**Affiliations:** Brighton and Sussex University Hospitals NHS Trust, UK

**Figure 1:**
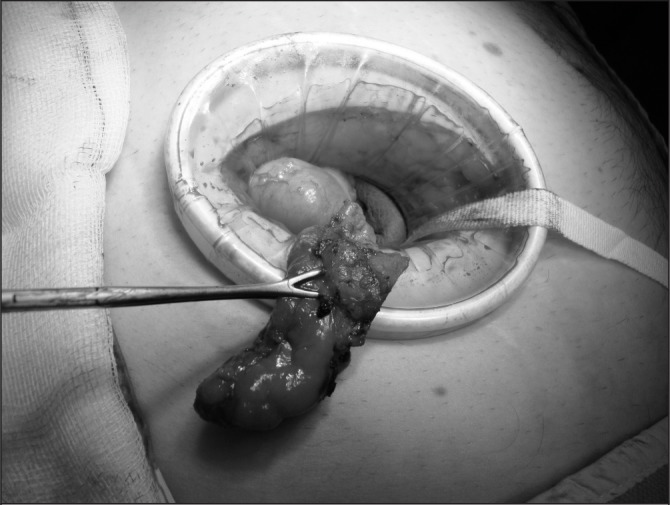
Alexis^®^ wound protector

Owing to the requirement of surgical senior house officers to prioritise acute admissions in the accident and emergency department, they are increasingly unable to be present in theatre to assist the surgical registrar. As a consequence, the surgeon has to rely on the scrub team to provide much-needed retraction with cases such as open appendicectomies. We have found the use of the Alexis^®^ wound protector (Applied Medical, London, UK) advantageous in this setting (Fig 1). It not only provides excellent wound retraction, reducing the need for an assistant, but also has the potential to reduce the incidence of surgical site infection.^1^
